# The genotypic and family characteristics and clinical intervention of neurofibromatosis type 1 gene are associated with dystrophic scoliosis by whole-exome sequencing

**DOI:** 10.3389/fneur.2025.1641665

**Published:** 2025-10-15

**Authors:** Yukun Du, Tianyu Bai, Jie Song, Li Zhang, Hui Huang, Peng Han, Fangfang Gai, Jianwei Guo, Jianyi Li, Changlin Lv, Jiale Shao, Guodong Zhang, Hao Tao, Yongming Xi

**Affiliations:** ^1^Department of Spinal Surgery, The Affiliated Hospital of Qingdao University, Qingdao, Shandong, China; ^2^Department of Health Care, The Affiliated Hospital of Qingdao University, Qingdao, Shandong, China; ^3^Department of Operation Room, The Affiliated Hospital of Qingdao University, Qingdao, Shandong, China; ^4^Department of Anesthesiology, The Affiliated Hospital of Qingdao University, Qingdao, Shandong, China; ^5^Qingdao-Europe Advanced Institute for Life Sciences, BGI-Qingdao, Qingdao, China

**Keywords:** neurofibromatosis type 1, dystrophic scoliosis, whole-exome sequencing, gene variants, surgical correction, family genetic characteristics

## Abstract

**Background:**

To investigate the genotypic and family genetic characteristics of neurofibromatosis type 1 (NF-1) patients associated with dystrophic scoliosis and to further evaluate the clinical efficiency of surgical intervention to these patients.

**Methods:**

A total of seven NF-1 patients with dystrophic scoliosis and their 11 immediate relatives were included in this study who visited The Affiliated Hospital of Qingdao University spinal surgery department from January 2020 to December 2022. The outcomes were summarized by investigating the clinical and imaging parameters before and after the treatment. Whole-exome sequencing (WES) was conducted to analyze the genotypic and family genetic characteristics of all patients and their families.

**Results:**

Among the seven patients, six patients underwent surgical treatment after follow-up. Compared to preoperative Cobb angle, the maximum postoperative correction rate was 85.3%. We identified eight pathogenic variants in the NF-1 gene. The variants c.c4084T and c. T4445C were located in the GAP-related domain, and the variant c. G1885A was shared by patient 3 and his diseased sibling. In the family of patient 3, variant c. G1885A was detected in both neurofibromatosis patients. The rs112819846 exhibited the most pronounced frequency disparity, whereas rs2916067 and rs80221306 were found in all patients.

**Conclusion:**

In this cohort study, NF-1 patients with dystrophic scoliosis predominantly presented with upper thoracic involvement, and surgical correction achieved a maximum postoperative Cobb angle correction rate of 85.3% without loss of correction during follow-up. The c. G1885A variant in the NF-1 gene may influence the phenotypic severity of the disease, while rs112819846, rs2916067, and rs80221306 may represent disease-relevant pathogenic variants of NF-1 patients with dystrophic scoliosis.

## Introduction

Neurofibromatosis is an autosomal dominant genetic disease with familial inheritance (1), with neurofibromatosis type 1 (NF-1) being the most common form of neurofibromatosis. NF-1primarily affects the peripheral nervous system (2), and often involves bones, skin, and soft tissues with an incidence of 10–60% (3). Approximately 50% of NF-1 patients are susceptible to bone malnutrition, with scoliosis being the highly prevalent bone damage (4). NF-1 scoliosis can be classified as dystrophic and non-dystrophic, with significant differences of natural history and treatment plans. Non-malnourished scoliosis, which represents the more common spinal deformity of NF-1 patients, resembles adolescent idiopathic scoliosis in terms of imaging manifestations, treatment plans, and complications (5). If the Cobb angle of the main curvature of this type of scoliosis exceeds 40°, posterior spinal fusion is required. Dystrophic scoliosis, the more severe subtype, usually presents with early onset, rapid progression, and distinctive radiographic features, including vertebral scalloping and rib penciling. Given its aggressive nature, timely surgical intervention and long-term management are essential to prevent severe deformity and related complications.

Previous studies have shown that the main cause of NF-1 is related to mutations of *NF-1* gene, leading to abnormalities in the encoding of neurofibromatosis proteins (6). The major reason for bone dysplasia in NF-1 is attributed to the reduced expression of neurofibromatosis proteins in osteoblasts, osteoclasts, chondrocytes, fibroblasts, and vascular endothelial cells (7). The *NF-1* gene is located on chromosome 17 (17q11.2), containing approximately 60 exons and spanning over 300 bases (8). Mutations types include nonsense, missense, insertion or deletion (frameshift), splicing, and complete gene deletion. Neurofibromatosis proteins are the gene product of NF-1, containing functional protein of 2,818 amino acids with molecular weight of 280kD. Neurofibromatosis proteins have homology between the 25–40 kDa fragment of NF-1GRD and the 250–400aa fragment of p120GAP, and act as GTPase-activating proteins (GAPs) on Ras to perform their primary function (9). GAPs are currently the only clear functional fragments of neurofibromatosis proteins. NF-1 mRNA is widely expressed in neural crest-derived tissues, bones, and vascular endothelium, and its protein product, neurofibromin regulates cell proliferation and differentiation mainly through the Ras/MAPK and PI3K/AKT signaling pathways. Moreover, specific NF-1 variants have been correlated with distinct clinical phenotypes, suggesting important genotype–phenotype associations in disease severity and manifestations.

Unlike adolescent idiopathic scoliosis, malnourished scoliosis is often accompanied by significant spinal abnormalities, such as acute angular deformity, vertebral rotation or subluxation, shortened ribs, and slender pedicle (10). The typical site is the upper thoracic spine, which is often accompanied by obvious shoulder imbalance. Early and active surgical intervention is necessary to prevent the progression of scoliosis in patients with neurofibromatosis. While previous studies have conducted comparative analyses of the surgical treatment, imaging manifestations, and other aspects of scoliosis in neurofibromatosis (11–14), there remains limited research on the intrinsic genetic initiating mutations and genetics of scoliosis in neurofibromatosis. Identification of specific pathogenic mutations and genetic characteristics in neurofibromatosis patients is of great significance for the prevention of scoliosis.

Whole-exome sequencing (WES) allows for the sequencing of more than 4,000 genes that currently have known biological functions. Exons, which comprise about 1.5% of the genome, account for approximately 85% of the pathogenic variants (15, 16). WES exhibit several benefits over other genomic techniques, including lower cost, higher throughput, and greater diagnostic potential. This method has wide coverage and can comprehensively screen for pathogenic genes related to skeletal developmental abnormalities. Research has shown that WES technology improves the diagnostic rate of fetal skeletal system diseases (17, 18). With decrease in sequencing costs, its clinical applications have become increasingly widespread in the recent years.

Therefore, this study conducted WES of neurofibromatosis patients with scoliosis, combined with relevant imaging data, to explore specific gene mutations and provide a theoretical basis for the prevention and treatment of scoliosis.

## Materials and methods

### Clinical specimen acquisition

This study enrolled seven NF-1 patients with dystrophic scoliosis who visited The Affiliated Hospital of Qingdao University spinal surgery department from January 2020 to December 2022. Two independent doctors diagnosed NF-1-associated dystrophic scoliosis based on established criteria. The diagnostic criteria were referenced as follows: (1) six or more café-au-lait spots ≥5 mm in diameter before puberty or ≥1.5 mm in diameter after puberty, (2) axillary or inguinal skinfold freckling, (3) two or more dermal neurofibromas or one plexiform neurofibroma, (4) two or more iris hamartomas (Lisch nodules), (5) an optic pathway glioma, (6) distinctive long bone dysplasia involving the sphenoid wing or thinning of the long bone cortex with or without pseudarthrosis, and (7) a first-degree relative with NF-1. NF-1 can be diagnosed if an individual presents with two or more of these symptoms (19). All patients who met the eligibility criteria were invited to participate in the study, and written consent was obtained from each participant to publish clinical and genetic data. The clinical data of all patients was also recorded. In addition, venous blood samples of patients and family members also collected for whole-genome sequencing to identify specific genes.

### DNA extraction

Blood samples were collected in ethylenediaminetetraacetic acid (EDTA) tubes and centrifuge them at 1,600 × g for 10 min at room temperature to separate the plasma. We used the MGIEasy Circulating DNA Isolation Kit (BGI) according to the manufacturer’s instructions. A total of 300 μL plasma samples were extracted by magnetic bead method. The extracted DNA was quantified using the Qubit Fluorometer (Invitrogen) and assessed for degradation and contamination via 0.8% agarose gel electrophoresis.

### Library preparation and sequencing

About 0.7 μg of total gDNA of each sample was firstly sonicated to 300–500 bp size range using the Covaris LE220 (Covaris). DNA fragments were then end-repaired, A-tailed, and purified. WES library preparation was performed with the MGIEasy Exome Capture V4 Probe following the manufacturer’s instructions. Pooled libraries were sequenced on the DNBSEQ-T1&T5 (PE 100) platform (BGI-Shenzhen), with a mean sequence coverage depth of 7 × for each tumor and NAT sample, respectively.

### Data preprocessing and alignment

FastQC v0.11.3 was conducted to qualify the raw sequencing reads. The low-quality reads were trimmed as clean reads using the fast (v0.19.6) software with default parameters. The remaining high-quality clean reads were mapped to the human reference genome (GRCh38) using BWA-MEM v0.7.17 and duplicate reads were marked using GATK MarkDuplicate (GATK v4.2.2.0). After alignment and trimming, all BAM files were sorted and indexed using SAMtools v1.15 for downstream processing analyses, including somatic copy number alteration (SCNA) and ecDNA analysis, somatic mutation, and structural variation detection.

### Somatic mutation calling, filtering, and annotation

Somatic mutations, including single nucleotide variants (SNVs) and insertions/deletions (Indels), were identified via local assembly of haplotypes by Mutect2, with the patient and matched normal BAMs as inputs (20). To ensure data reliability, we applied multiple quality control measures, including sequencing depth ≥100 × for targeted regions, minimum base quality score ≥30, mapping quality ≥50, and variant allele frequency (VAF) threshold ≥0.05. FilterMutectCalls in GATK (v4.2.5.0) was used to filter the raw output of Mutect2, removing sequencing artifacts and low-confidence calls. Candidate variants were further evaluated by excluding common polymorphisms using population databases (gnomAD, 1,000 Genomes, ExAC) and annotated using ANNOVAR. In addition, we manually inspected variants with Integrative Genomics Viewer (IGV) to confirm their authenticity. This systematic pipeline, integrating variant calling, multi-level filtering, and functional annotation, has been widely validated in previous studies (21, 22).

### Mutational signature analysis

We excluded non-synonymous variants that were shared by all family members. We then removed variants with a population frequency above 0.01 (23). Lastly, we annotated the variants with their clinical significance and identified the pathogenic variants among all family members using the ClinVar database, which is a public repository that collects and disseminates data on genomic variants, their clinical relevance, and supporting evidence (24). We assigned 18 participants to either the patient group (seven probands and one relative) or the control group (10 non-diseased family members) according to their neurofibromatosis status. Differentially pathogenic variants between the patient and control groups were identified using a one-sided Fisher’s exact test, with statistical significance defined as *p* < 0.05. This test was selected because of the small sample size and categorical nature of the variant frequency data.

## Results

### Baseline characteristics of the participants

Three male patients and four female NF-1 patients with dystrophic scoliosis were enrolled in this study (Table 1). The range of age was from 10 years to 17 years. A total of six patients’ immediate family also underwent venous blood sampling for comprehensively analyzing the specific pathogenic genes caused by neurofibromatosis. Abnormalities of thoracic vertebrae were detected in five of seven patients. Among the seven patients, three had thoracic scoliosis (including one with kyphotic deformity), two had lumbar scoliosis, and two had thoracolumbar involvement. The apical vertebrae were mainly located in the upper thoracic region (T4–T12), and two patients presented with shoulder imbalance. One patient (patient 3) showed complex vertebral anomalies, including intravertebral rib head, hemivertebra, and abnormal pedicles. Family history was positive in two patients, with affected first-degree relatives.

**Table 1:** Clinical information of 7 NF-1 patients with dystrophic scoliosis.

ID	Sex	Age	Duration	Type scoliosis	Shoulder imbalance	Apical vertebrae	Cobb at first attendance	Preoperative Cobb	Last follow-up Cobb	Last follow-up correction rate (%)	Surgery	Fixation segment	Family history	Spine abnormalities
1	F	13 years	1 year	LTS	No	T11	-	57°	6.5°	88.6%	Yes(PSF)	T4-L1	None	None
2	M	10 years	1 year and 4 months	RTS	Yes (3.2°)	T5	28°	47.5°	8.8°	81.5%	Yes (PSF-Robot assistant)	T2-T8	His mother	None
3	M	13 years	3 months	kyphotic deformity	Yes	T4	-	36.9	6.5°	82.4%	Yes(PSF)	T4-L1	His brother	Intraspinal rib head(T9, T10); Hemivertebra(T9); abnormal pedicles(T9, T10)
4	F	16 years	1 year and 6 months	RTS	Yes	T8	-	45°	7.5°	83.3%	Yes(PSF)	T3-12	None	None
5	M	17 years	2 months	RTS	Yes (3.3°)	T6	-	68°	7.9°	85.3%	Yes(PSF)	T2-10	None	None
6	F	15 years	3 years and 10 months	LLS	No	T12	11°	56°	13°	76.8%	Yes(PSF)	T6-L4	Mother and sister	None
7	F	15 years	1 year	RLS	No	L2	9°	-	-	-	No	-	None	None

LTS, Left thoracic scoliosis; RTS, Right thoracic scoliosis; LLS, Left lumbar scoliosis; RLS, Right lumbar scoliosis; PSF, Posterior spinal fusion.

Among the seven patients, the shortest and longest follow-up time are 6 months and 2 years, respectively, since the diagnosis of scoliosis. Ultimately, six patients underwent surgical treatment after follow-up and there were no related complications such as increased Cobb angle, failed internal fixation, or neurological dysfunction.

Seven neurofibromatosis patients underwent regular X-ray from the first diagnosis to determine the progression of scoliosis. Among the six surgical patients, the minimum and maximum Cobb angle were 36.9° and 68°, respectively, before surgery. Compared to preoperative Cobb angle, the maximum postoperative correction rate was 85.3%. One patient had abnormal vertebral development, including small pedicle, rib head invasion into the vertebral canal, and hemivertebra. Among the six patients who underwent surgery, the longest fixed segment spanned 10 vertebral levels (T6-L4). Postoperative follow-up of all patients showed no loss of correction (Table 1).

### Identification of pathogenic variants of NF-1 gene

**Figure 1:**
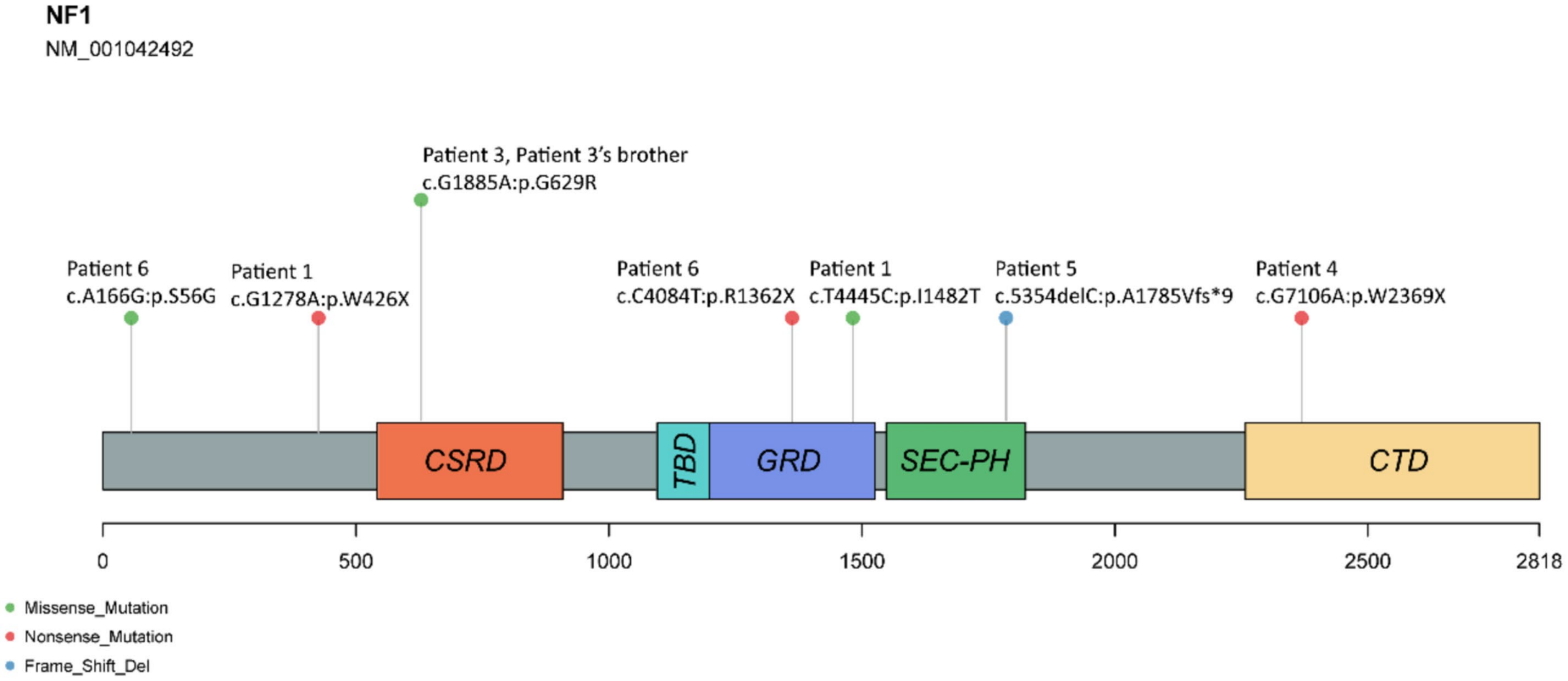
Schematic representation of distribution of variants in NF-1 and neurofibromin domains. The amino acid number is indicated below. CSRD, cysteine- and serine-rich domain; TBD, tubulin-binding domain; GRD, GAP-related domain; Sec-PH, Sec14 homologous and pleckstrin homologous domain; CTD, C-terminal domain. The amino acid number is indicated below.

We performed WES on seven patients and their 11 immediate relatives, and identified eight pathogenic variants in the *NF-1* gene after filtering and annotation. Of these, seven variants were present in neurofibromatosis patients (five patients and sibling of patient 3). Only c.8413A > T was unique to unaffected family members. All of these identified variants were previously reported in public databases and are not novel. We examined the association between amino acid positions altered by *NF-1* variants in patients and peptide chain domains (Figure 1). We discovered that variants c.4084C > T and c.4445 T > C are located in the GAP-related domain (GRD), which is a well-studied domain. We also observed that variant c.1885G > A, which was present in the patient exhibiting abnormal vertebral morphology, resided in the cysteine- and serine-rich domain (CSRD) (Table 2).

**Table 2:** Variants of gene NF1 in 7 probands and their immediate relatives.

ID	NF-1 status	Detection method	Gene	Variants classification	Variants	AAchange	Classification
Patient 1	Disease	WES	NF1	Missense_Mutation	Exon33:c. T4445C	p. I1482T	Pathogenic
				Nonsense_Mutation	Exon12:c. G1278A	p. W426X	
Patient 1’s father	Normal	WES	NF1	Missense_Mutation	Exon33:c. T4382C	p. I1461T	Pathogenic
Patient 2	Disease	WES	NF1	-	-	-	-
Patient 2’s father	Normal	WES	NF1	-	-	-	-
Patient 2’s mother	Normal	WES	NF1	-	-	-	-
Patient 3	Disease	WES	NF1	Missense_Mutation	Exon17:c. G1885A	p. G629R	Pathogenic
Patient 3’s brother	Disease	WES	NF1	Missense_Mutation	Exon17:c. G1885A	p. G629R	Pathogenic
Patient 3’s mother	Normal	WES	NF1	-	-	-	-
Patient 4	Disease	WES	NF1	Nonsense_Mutation	Exon47:c. G7106A	p. W2369X	Pathogenic
Patient 4’s sister	Normal	WES	NF1	-	-	-	-
Patient 4’s father	Normal	WES	NF1	-	-	-	-
Patient 4’s mother	Normal	WES	NF1	Nonsense_Mutation	Exon47:c. G7043A	p. W2348X	Pathogenic
Patient 5	Disease	WES	NF1	Frame_Shift_Del	Exon37:c.5354delC	p. A1785Vfs*9	Pathogenic
Patient 5’s father	Normal	WES	NF1	-	-	-	-
Patient 5’s mother	Normal	WES	NF1	Missense_Mutation	Exon57:c. A8413T	p. S2805C	Pathogenic
Patient 6	Disease	WES	NF1	Missense_Mutation	Exon2:c. A166G	p. S56G	Pathogenic
				Nonsense_Mutation	Exon30:c. C4084T	p. R1362X	
Patient 7	Disease	WES	NF1	-	-	-	-
Patient 7’s mother	Normal	WES	NF-1	-	-	-	-

All participants performed Whole Exome Sequencing (WES), The reference genome assembly was GRCh38, the clinical features were obtained from the ClinVar database, and the gene annotation was performed using ANNOVAR. AA, amino acids; WES, Whole Exome Sequencing; Del, deletion.

### Family-based analysis

**Figure 2:**
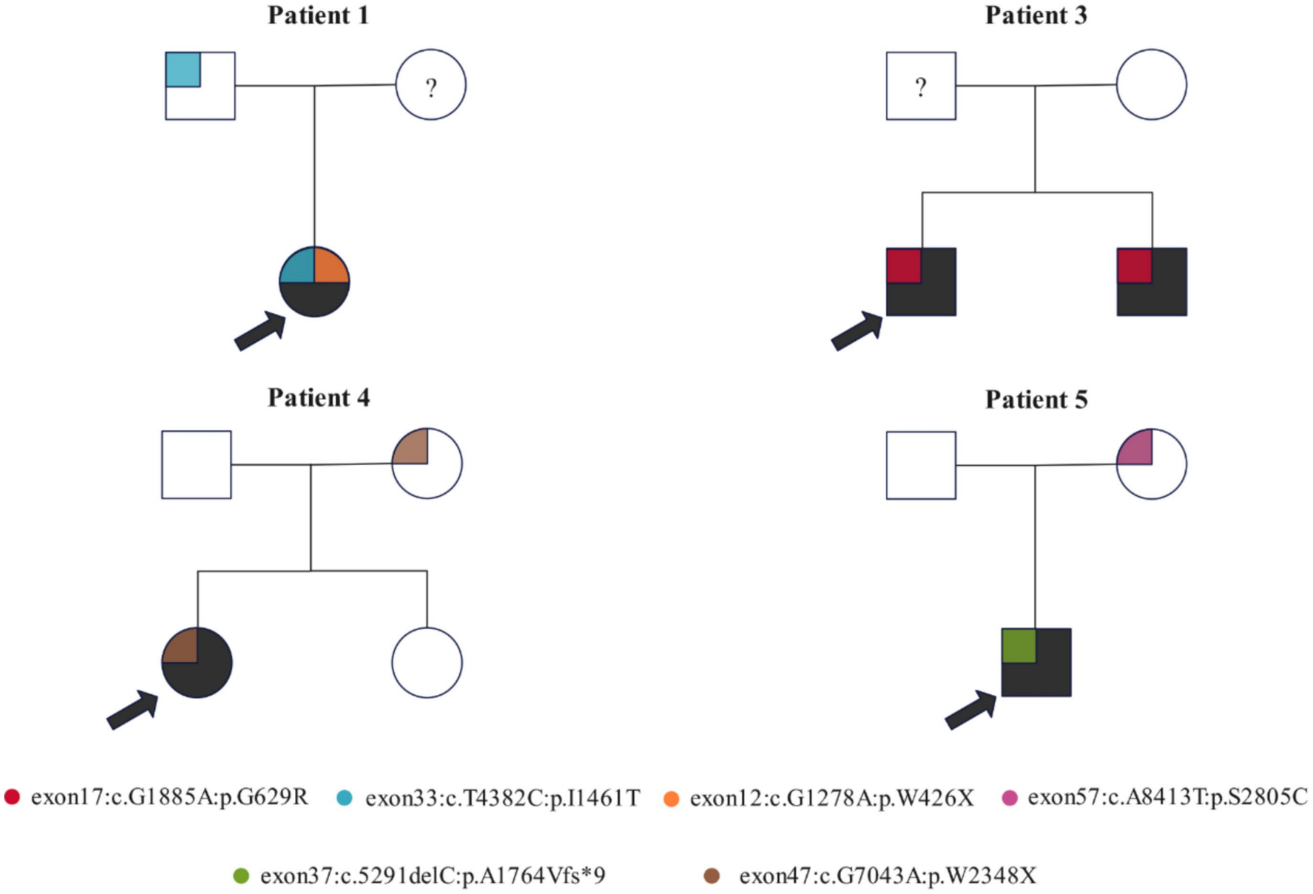
Family-based analysis of the genetic variants of the NF-1 gene in four probands and their families. Variants are represented by blocks of different colors. The pedigree symbols are as follows: square for male, circle for female, arrow for proband, “?” for specimen unavailable, and shaded for NF-1 diseased.

Family-based analysis was performed in the families of four patients with *NF-1* gene variants (Figure 2). Patient 1 inherited c.4382 T > C from her father. Moreover, due to the unavailability of the mother’s sample, the origin of variant c.1278G > A remains undetermined. In the family of patient 3, variant c.1885G > A was detected in both neurofibromatosis patients, suggesting that it may influence the penetrance of neurofibromatosis. In the family of patient 4, variant c.7043G > A was present in the patient and her non-diseased mother. Finally, as reported in patient 5’s family, c.5291delC was associated with the penetrance of *NF-1*.

### Identification of disease-related variants

We performed a one-sided analysis of the pathogenic variants that are associated with the disease phenotype in the patient and control groups, and we detected five disease-related variants with statistically significant frequency disparities. All these variants were loci with high mutation rates that are cataloged in the dbSNP database. Among them, rs112819846 exhibited the most pronounced frequency disparity (*p* = 0.00653), whereas rs2916067 (*p* = 0.01282), and rs80221306 (*p* = 0.0359) were found in all the patients with NF-1 (Table 3).

**Table 3:** NF1-related mutations with significant differences by one-sided fisher’s exact test.

ID	Gene	Chromosome	Variant classification	Variants	AAchange	Patient (*n* = 8)	Control (*n* = 10)	*p* value
rs112819846	MAGEF1	chr3	In_Frame_Ins	exon1:c.476_477insGGA	p. E158_D159insE	5 (62.5%)	0 (0%)	0.00653
rs2916067	FCGBP	chr19	Missense_Mutation	exon17:c. A8174C	p. N2725T	8 (100%)	4 (40%)	0.01282
rs771265921	LILRB3	chr19	Substitution	exon6:c.1198_1199delinsTA	R400_A630delinsYSSN*	5 (62.5%)	1 (10%)	0.04298
rs79483025	LOC100996413	chr15	Missense_Mutation	exon2:c. C416T	p. P139L	6 (75%)	1 (10%)	0.01282
rs80221306	FLG	chr1	Missense_Mutation	exon3:c. A8807G	p. D2936G	8 (100%)	5 (50%)	0.0359

The dbSNP identifiers (IDs), the reference genome assembly GRCh38, the ClinVar database for clinical characteristics, and the ANNOVAR software for gene annotation were used in this study. AA, amino acids; del, deletion; ins, insertion.

## Discussion

In 1987, the National Research Institute of the United States proposed seven diagnostic criteria for NF-1, including skin milky coffee spots, neurofibromas, inguinal freckles, optic gliomas, Lisch nodules, characteristic skeletal change, and family history (25). Due to some diagnostic features changing with age, the international NF-1 diagnostic standard has some limitations, which may lead to delayed diagnosis in some patients. Spinal scoliosis, as the most common bone system lesion in NF-1, can be diagnosed on changes in patient’s physical appearance clinical examination, typically presenting during the first peak period between 5 and 10 years of age (26). According to previous studies, the incidence of NF spinal deformities is 10–64% with the faster progression rate of deformities than those of AIS (27). Therefore, scoliosis screening should be performed in all pre-adolescent children with NF-1. Due to the significant differences in treatment and prognosis between the two types of scoliosis associated with NF-1, it is vital to make timely diagnosis on the types of scoliosis.

Patients with NF-1 accompanied by scoliosis can be divided into malnourished and non-malnourished based on imaging features. Non-malnourished scoliosis typically presents with long, smooth curves. In contrast, malnourished scoliosis, compared with AIS, typically involves four to six vertebral bodies and is characterized by short and sharp lateral curvature, with or without angular kyphosis. In addition, the undernourished scoliosis, which often develops gradually in young age, is accompanied by pencil-shaped changes of ribs, vertebral rotation, vertebral wedge-shaped changes, and lateral curvature of atypical positions (28). Li et al. measured 2,652 pedicle abnormalities of 56 NF-1 patients with dystrophic scoliosis and 22 NF-1 patients with non-dystrophic scoliosis (29). The results showed that the overall incidence of pedicle abnormalities in NF-1 patients was as high as 67%, and the upper thoracic spine (87%) was the most concentrated area of abnormal pedicle abnormalities. In addition to scoliosis, patients with neurofibromatosis often have abnormalities in the skin, blood vessels, eyes, and nervous system. Approximately 20–50% of NF-1 patients have osteoporosis, which is associated with alterations in bone transformation biochemical markers, including decreased serum 25-hydroxyvitamin D and elevated serum PTH levels (30). In this study, one patients also experienced changes in multiple imaging indicators, such as intraspinal rib head(T9, T10),a hemivertebra(T9,)and abnormal pedicles(T9, T10). The accurate recognition of these radiological features is beneficial for early diagnosis of patients with malnourished scoliosis to develop timely and appropriate treatment plans.

With the development of modern diagnostic technology, NF-1 can be diagnosed early through genetic testing. In 2021, the International Consensus Group of Diagnostic Standards for Neurofibromatosis proposed revisions of the NF-1 diagnostic standard (1987) mainly incorporating genetic diagnosis (31). Genetic testing was able to confirm diagnosis before clinical diagnosis (or before the appearance of the second clinical feature), which could help screen out children with atypical features. The NF-1 gene has been recognized as the pathogenic factor for NF-1 in the medical community. However, the correlation between different mutation types and sites of NF-1 and the clinical phenotype of NF-1 patients has always been a hot research topic in the medical community. Although scoliosis is the most common type of skeletal changes in NF-1, there is limited research on the correlation between specific mutation types and mutation sites in NF-1 patients with scoliosis.

In this study, we conducted WES on 18 individuals from seven probands and their immediate relatives to explore gene variants associated with NF-1. As previous studies have shown that mutations in the NF-1 gene lead to the occurrence of NF-1, we performed family-based analysis to identify the variants of NF-1 gene associated with phenotypes and characterized their effects on the amino acid sequence and structure (32). The NF-1 gene codes for neurofibromin, a protein of 2,818 amino acids that negatively regulates the RAS pathway, a signaling cascade involved in cell growth and differentiation (33). Neurofibromin acts as a GAP for Ras, a family of small GTP-binding proteins that are key regulators of the RAS pathway. A 360 amino-acid segment of neurofibromin, called the GAP-related domain (GRD), enhances the GTP hydrolysis of Ras, thereby inactivating it (34). *NF-1* mutations impair the function of neurofibromin, resulting in the accumulation of active Ras and the hyperactivation of the RAS pathway, which underlies the tumorigenesis and other phenotypes of *NF-1*. We identified two mutations located in the GRD region in patients 1(c.4445 T > C, p. Ile1482Thr) and 6(c.4084C > T, p. Arg1362Ter). c.4445 T > C is a single nucleotide variation located at chr17:31260383 with a minor allele frequency of 0.000024, resulting in a missense mutation of isoleucine to threonine at codon 1,482. This SNV is associated with not only NF-1 (NF-1) but also hereditary cancer predisposition syndrome. c.4084C > T is located at chr17:31249093 with a minor allele frequency of 0.000008, resulting in a premature stop codon in the mRNA transcript and leading to a truncated protein product. Eisenbarth et al. (35) identified c.4084C > T as a tumor-specific point mutation in seven neurofibromas from four different NF-1 patients and demonstrated that loss of function of NF-1 gene is a common mechanism in tumorigenesis. Hence, variants in the GRD may impair the function of neurofibromin and contribute to the genetic predisposition for neurofibromatosis.

In the family-based analysis, we identified c.1885G > A in the NF-1 gene in patient 3 and his sibling, both of whom exhibited NF-1 phenotypes, and this variant was absent in their normal parents. Furthermore, patient 3 uniquely presented with vertebral body dysplasia, indicating that c.1885G > A is a genetic factor that contributes to the development of NF-1. c.1885G > A is an SNV at chr17:31249093 with a minor allele frequency of 0.000024, resulting in a nonconservative amino acid substitution in the CSRD of neurofibromin. The CSRD of neurofibromin undergoes phosphorylation by protein kinase A (PKA) and protein kinase C (PKC). The PKC-mediated phosphorylation of the CSRD modulates the conformation and function of neurofibromin, a Ras-GAP, and promotes its interaction with actin. In our cohort study, none of the studied families had consanguineous marriage, suggesting that the identified variants were not attributable to consanguinity.

Through a one-sided analysis between the patient and control group, we detected five NF-1 related pathogenic variants with statistically significant frequency. We observed that two variants, rs2916067 and rs80221306, were present in all the individuals with NF-1 in our study. Variant rs2916067 is an SNV in the coding region of MAGEF1, a ubiquitin ligase enhancer and a member of the melanoma-associated antigen (MAGE) super family (36). MAGEA2 is overexpressed in various cancers and suppresses the RAS pathway. Correspondingly, loss-of-function mutations in the NF-1 gene inhibit the RAS pathway, impair bone development, and lead to skeletal deformities such as scoliosis and pseudarthrosis. Variant rs80221306 is located at gene filaggrin (FLG), which affects differentiation of keratinocytes (37). The dysregulation of the keratinocyte–melanocyte axis results in the formation of café-au-lait macules and malignant melanoma in NF-1, but the underlying molecular basis of this dysregulation remains elusive. The FLG gene might play a role in facilitating phenotypic expression.

There are still some limitations that need to be addressed. First, the small sample size of this study reduces the statistical power. However, the NF-1 is quite rare condition, and only a few of patients present with the dystrophic scoliosis. In addition, WES is an expensive test for patients, which limit the sample size. Second, all participants in the present study were from a single ethnic group, which limits the generalizability of the findings to the broader human population. Third, the study would have been more robust if genetically healthy humans were considered as the control group. Another limitation of this study is that we did not perform functional assays to validate the biological effects of the identified NF-1 variants, and further experimental studies are required to confirm their pathogenicity.

## Conclusion

In our cohort study, NF-1 patients with dystrophic scoliosis predominantly exhibited upper thoracic involvement, and surgical correction achieved satisfactory outcomes, with a maximum postoperative Cobb angle correction rate of 85.3% and no loss of correction during follow-up. The c. G1885A variant in the NF-1 gene may influence the phenotypic severity of the disease, and rs112819846, rs2916067, and rs80221306 may be the disease-related pathogenic variants of NF-1 patients with dystrophic scoliosis.

## Data Availability

The raw data supporting the conclusions of this article will be made available by the authors, without undue reservation.
